# Bone Fracture Incidence in Postmenopausal Women: Results of a 10 Year Follow Up in a RAC-OST-POL Study of rs1544410, rs7975232 and rs731236 Polymorphisms

**DOI:** 10.3390/nu16234146

**Published:** 2024-11-29

**Authors:** Elżbieta Tabor, Sylwia Górczyńska-Kosiorz, Wojciech Pluskiewicz, Janusz Gumprecht

**Affiliations:** 1 Department of Internal Medicine, Diabetology and Nephrology Faculty of Medical Sciences in Zabrze, Medical University of Silesia, 40-055 Katowice, Poland; skosiorz@sum.edu.pl (S.G.-K.); jgumprecht@sum.edu.pl (J.G.); 2 Metabolic Bone Diseases Unit, Department of Internal Medicine, Diabetology and Nephrology Faculty of Medical Sciences in Zabrze, Medical University of Silesia, 40-055 Katowice, Poland; wpluskiewicz@sum.edu.pl

**Keywords:** bone fracture, fracture risk, genetic polymorphism, genotyping, long-term follow up, paternal, postmenopausal osteoporosis, VDR

## Abstract

Background: The clinical significance of the genetic influence of vitamin D receptor polymorphisms has still not been well-analyzed. Objectives: To verify whether rs1544410, rs7975232 and rs731236 polymorphisms are associated with a higher 10-year fracture risk in postmenopausal women. Methods: The study group was a subset of a pre-defined population as part of the broader epidemiological research called the RAC-OST-POL Study and consisted of 358 postmenopausal women, chosen randomly from Racibórz (Poland) inhabitants (mean baseline age 65 ± 6.9 years, BMI 31.2 ± 5.5 kg/m^2^). From all participants’ medical history, data concerning co-morbidities, fracture history, the medication used, parental history of bone fractures, cigarettes and alcohol use were taken at baseline. Moreover, rs1544410, rs7975232 and rs731236 polymorphisms were analyzed. Next, over the following 10 years, participants were contacted once a year and questioned concerning new fractures events and their circumstances. Results: We did not find statistically significant main effects on the fracture incidence of single-polymorphism variants. However, there were some significant findings dependent on the co-existence of these polymorphisms and medical factors. Women with a positive history of parental fracture and configuration of CC rs7975232, AA rs731236 and CC rs1544410 had a higher fracture incidence. The risk of bone fracture was also significantly higher in the group of heterozygotes of AC rs7975232 if their BMI value was in the categories of normal weight or overweight, or if they were treated with calcium or vitamin D. Conclusions: Polymorphisms of rs1544410, rs7975232 and rs731236 are connected with the fracture incidence in postmenopausal women. Nevertheless, its influence should be considered with co-existing clinical factors, especially paternal fracture history, prior fracture, BMI value, any osteoporotic treatment or calcium/vit. D supplementation.

## 1. Introduction

Osteoporosis is a disease characterized by reduced bone mineral density, which makes it more susceptible to fractures, mainly femoral neck, forearm and vertebral fractures. Postmenopausal women are particularly at a risk of developing osteoporosis. Very often, osteoporosis is diagnosed only after the first fracture. According to epidemiological statistics for the year 2019, as many as 32 million people over 50 years of age suffer from osteoporosis in Europe [1]. It is estimated that by the year 2050 the incidence of hip fractures worldwide may increase by 310% and 240% among men and women, respectively, vs. the data from 1990 [2]. According to European statistics, fragility fractures are the fourth leading cause of chronic diseases, behind ischemic heart disease, dementia and lung cancer [3]. Osteoporosis and osteoporosis-related bone fractures inflict enormous economic, social and health costs [4,5]. Moreover, fracture treatment and post-treatment physiotherapy generate much higher costs than fracture prevention [6]. The main goals of osteoporosis diagnosis and treatment include fracture prevention and the precise identification of the risk group.

There are many well-known fracture risk factors [7,8]. Some of them cannot be changed, i.e., age, genes, gender. Still, the awareness which of them predispose to a higher fracture risk may help implement effective fracture preventions programs.

A better understanding of the genetic pathways of osteoporosis may help identify a new therapeutic paradigm for patients with osteoporosis. The best known factors of bone loss to date include estrogen receptor α (ERα) and vitamin D receptor (VDR) [9]. The VDR gene is located on the long arm of chromosome 12 (12q13.11), consists of 11 exons and includes about 75 kb. Exons 1A, 1B and 1C are located on the noncoding 5′-prime end and the translated product is encoded by exon 2 to exon 10 [10]. There are many polymorphisms found in the VDR gene, including rs1544410 (called BsmI) and rs7975232 (ApaI) laying in intron 8, and rs731236 (TaqI; exon 9). The variant rs731236 (NM_000376.3):c. 1056T>C p.(Ile352=) is synonymous [11]; the other intron variants are as follows: rs1544410 (N M_000376.3): c.1024 +283G>T [12]; and rs7975232 (NM_000376.3): c.1025-49G>T [13], and may be responsible for the stability of the resulting mRNA [14].

The current literature reveals many opposite observations concerning the impact of these single-nucleotide polymorphisms (SNPs) on the bone mineral content [14,15,16,17,18,19]. This may be caused by ethnic diversity and genetic heterogeneity. There is also an intriguing question whether the specific gene pattern may initially indicate candidates for the screening examinations toward earlier osteoporosis, or more intensive treatment and vitamin D/calcium supplementation [20]. As most research projects on VDR polymorphisms focus on bone parameters (mainly bone mineral density) [17,18,19,21], there is a lack of studies evaluating the real clinical risk of fracture. This rather inconsistent state of knowledge regarding the VDR polymorphism correlation with bone fracture risk was one of the inspirations for our study project. Our primary goal was to verify whether rs1544410, rs7975232 and rs731236 polymorphisms were associated with a higher 10-year fracture risk in postmenopausal women. The null hypothesis was that the population is at the Hardy–Weinberg equilibrium (HWE).

## 2. Materials and Methods

### 2.1. Study Design and Patient Selection

The study, approved by the Bioethical Committee of the Medical University of Silesia (no. KNW/0022/KB1/9/I/10, approval date 9 January 2010) was a part of the epidemiological project called the RAC-OST-POL study [22]. The participants of that project were randomly recruited from the general female population, aged over 55 years (the mean menopausal age in the Polish population is 49 years), inhabiting the province of Racibórz, a town in the Silesian Province in southern Poland. Invitations were sent via regular post to 1750 subjects (10% of population in the abovementioned gender and age group) and was responded to by 625 women, all of them with at least one year after the last menstruation. All the eligible women gave informed consent to participate in the study.

Medical history, including co-morbidities, previous fracture records, the medications used, the parental history of bone fractures, and cigarette and alcohol abuse, was obtained from all the participants. Next, body weight and height were measured, and the BMI was calculated according to the WHO criteria for each participant [23]. The initial part of the study took place in May 2010. During the 10 subsequent years, each of the participants was contacted and asked about any low-trauma fracture in the last 12 months, its circumstances and the number of bones fractured.

Only those participants who had remained in the 10-year survey were included in the analysis. The final sample consisted of 358 female patients. All the participants declared their postmenopausal status, defined in the study as the absence of menses for more than 1 year (the mean age of menopausal women was 48.9 years).

### 2.2. Genomic DNA Isolation and Polymorphism Genotyping

The isolation of genome DNA and polymorphism evaluation took place at our laboratory of the Department of Internal Medicine, Diabetology and Nephrology, Medical University of Silesia, Zabrze, Poland. Venous blood samples were collected from each participant and frozen in a temperature of −20 °C until DNA isolation. Genome DNA was isolated from leukocytes with the use of a special isolation kit from the Epicentre Technologies Company. The genotyping of polymorphisms was carried out using fluorescently labeled probes of TaqMan Assay kits (Applied Biosystems) for SNPs rs1544410, rs7975232 and rs731236 (see Supplementary Table S1). Each of the primary-group participants underwent the genotyping stage but, in some of them, the obtained results were incomplete for any of the above-mentioned polymorphisms. The complete results (all planned genotypes assessed) were achieved in 603 examined women.

### 2.3. Latent-Class Analysis for Polymorphism Patterns

**Figure 1:**
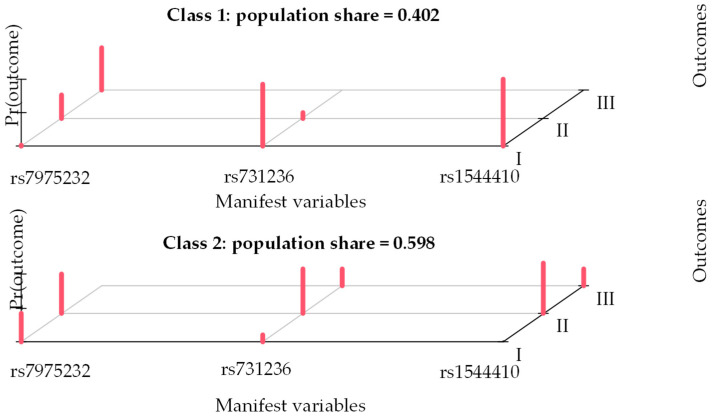
Characteristics of the groups extracted with latent-class analysis regarding the co-occurrence of polymorphism pattern variants. Note: Figure depicts what is the probability that each of the three polymorphisms is present in the two extracted groups. Legend: for rs7975232: I—AA, II—AC, III—CC; for rs731236: I—AA, II—AG, III—GG; for rs1544410: I—CC, II—CT, III—TT.

In order to extract the group of patients with a co-occurrence of similar patterns, regarding variants of examined polymorphisms, a latent-class analysis was performed. Two groups of patients were extracted. The fit indices were equal to AIC = 1697.90, BIC = 1748.35 and G2 = 257.42. The results are presented in Figure 1.

The first group (Group 1, *n* = 144) included patients with the co-occurring CC genotype of rs7975232, AA of rs731236 and CC of rs1544410. The second group (Group 2, *n* = 214) included patients with the co-occurring AC genotype of rs7975232, AG of rs731236 and CT of rs1544410.

### 2.4. Statistical Analysis

All statistical analyses were carried out by the Statistica 13.1 (StatSoft) software package. Data distribution was checked with the use of the Shapiro–Wilk test. The data with normal distribution were presented as the mean values ± standard deviations, whereas those with a non-normal distribution were expressed as the means of medians and quartiles. The data with a non-normal distribution were analyzed using the Spearman rank test, while the U Mann–Whitney and Kruskal–Wallis tests were applied for the analysis between two or more groups. The data with a normal distribution were analyzed with the use of *t*-tests. T-scores were used in the correlation analysis minus the values. *p*-values below 0.05 were assumed to be statistically significant.

Data analysis was performed in three stages. First, descriptive statistics were calculated. Based on the distribution of the variables, appropriate clinical characteristics were included in further analyses as moderators. Second, the aforementioned latent-class analysis allowed for the comparison of the genetic patterns regarding bone fractures. In the main analysis, the associations between specific variants of polymorphisms and bone fractures were analyzed with the use of binary regression analyses. The main effects of the variants and interaction effects between the variants of polymorphisms and a set of clinical characteristics were verified. The main purpose was to identify the variants associated with a higher risk of bone fractures in specific groups of patients, defined by their clinical characteristics. The acquired interaction effects were further interpreted on the basis of simple effect analyses. Odds ratios (ORs) were used as the measure of association. OR values above 1 indicated a higher risk for the event analyzed, i.e., in this particular case, at least one bone fracture during the previous 10 years. OR values below 1 indicated a lower risk for the events analyzed.

The relationships between polymorphism variants, medical characteristics and bone fractures were verified with the use of logistic regression analysis. The polymorphism variants were analyzed as explaining variables, medical characteristics as potential moderators and the dichotomous variable containing the information—if a patient had at least one bone fracture record in the 10-year follow up or not—as the explained variable. The following variants were analyzed as reference categories with the use of dummy coding: AA of rs7975232, GG of rs731236 and TT of rs1544410, i.e., the alternative variants in the population. In the analyses involving the extracted classes (see Figure 1), the group of patients with co-occurring CC rs7975232, AA rs731236 and CC rs1544410 was analyzed as a reference category (Group 1). Regarding the clinical characteristics, the cases of chronic alcohol use and calcitonin therapy were excluded from the analysis due to the small number of participants. The main effects of polymorphism variants were analyzed to verify if they increased or reduced the risk of bone fracture in general. The interaction effects between the polymorphism variants and the clinical characteristics were analyzed to determine if the effects of the former were modified, depending on the patients’ medical characteristics.

## 3. Results

### 3.1. Participants

The descriptive initial characteristics of the final study group are presented in Table 1.

**Figure 2:**
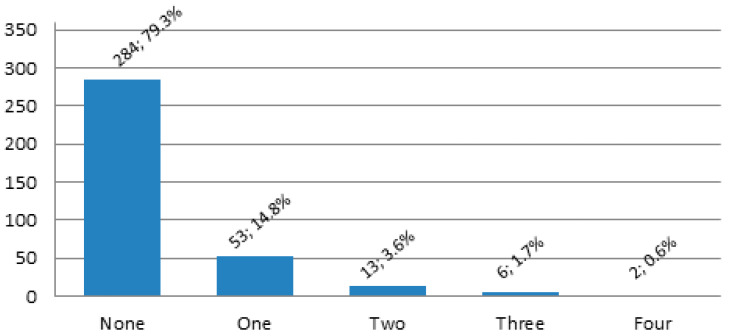
The number of bone fractures in the 10-year observation.

**Figure 3:**
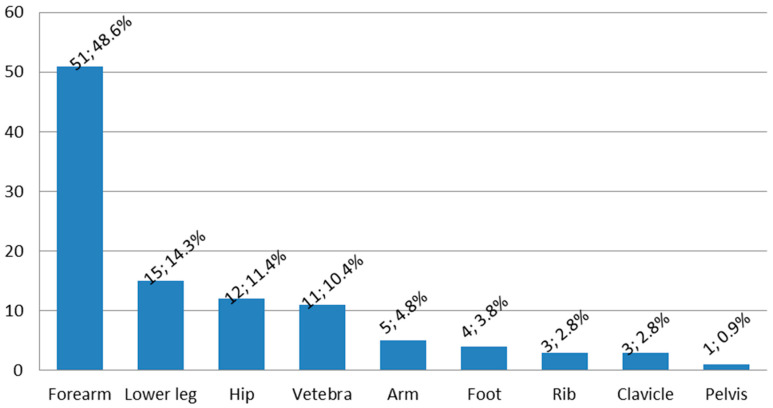
Fracture locations.

As many as 106 participants (29.6%) experienced any bone fracture prior to the study onset. Out of 358 patients, 74 (20.7%) had at least one bone fracture incident in the following 10 years. Figure 2 and Figure 3 shows the distribution of the bone fracture prevalence rates and locations in the 10-year follow up of the analyzed sample.

### 3.2. Polymorphism Variants

Three SNPs were examined. Table 2 presents the prevalence rates for the distribution of genotypes and alleles with values of the Chi-square test for a hypothesis that the population is at the Hardy–Weinberg equilibrium (HWE).

### 3.3. Polymorphism Variants as Risk Factors for Bone Fractures

The results acquired, i.e., the values of the odds ratios and *p*-values, are presented in Table 3.

**Figure 4:**
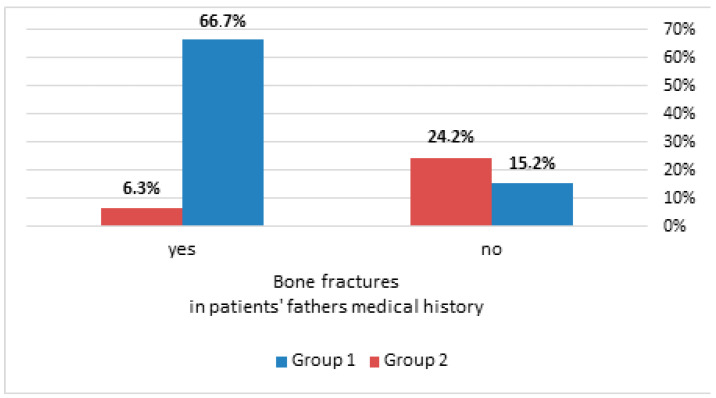
The distribution of bone fractures in the last 10 years in the extracted classes of co-occurring polymorphism variants in the group of patients with a bone fracture in their father’s medical history and in the group of patients without a bone fracture in their father’s medical history.

The results of the simple effects analysis revealed that the risk of a bone fracture was significantly higher for Group 2 than in Group 1, OR = 1.78, *p* < 0.05, but only in patients with a negative paternal history of bone fractures (negPBF). Regarding patients with a positive paternal history of bone fracture (posPBF), the opposite was true, i.e., the bone fracture risk was significantly lower in Group 2 than in Group 1: OR = 0.03, *p* < 0.05 (see Figure 4).

**Figure 5:**
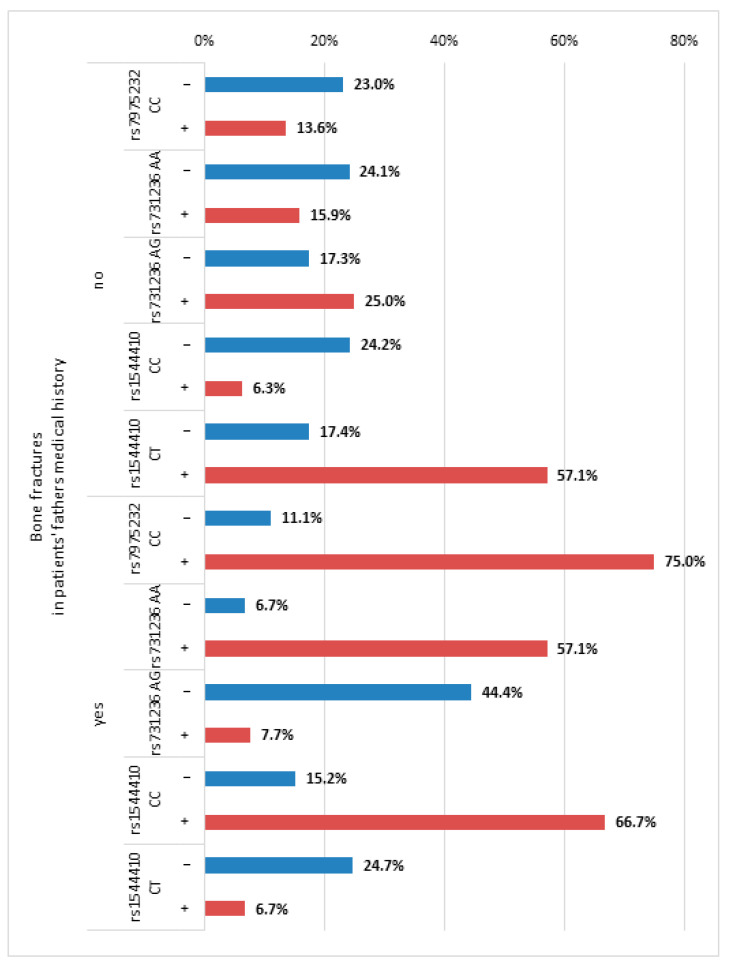
The distribution of bone fractures in the last 10 years depending on the polymorphism variants in the group of patients with a bone fracture in their father’s medical history and in the group of patients without a bone fracture in their father’s medical history. Note: the figure depicts in what percentages of participants with each polymorphism there was a bone fracture in patients’ fathers’ medical history. Legend: “+” polymorphism present; “−“ polymorphism absent.

The bone fracture risk was also significantly higher in the group of patients with alleles CC of rs7975232, OR = 24.00, *p* < 0.05, but only in patients with posPBF (see Figure 5). The risk of bone fracture was significantly higher in the group of patients with AA rs731236, OR = 18.67, *p* < 0.05; that effect was observed only for patients with posPBF. The risk of bone fractures was lower in the group of women with a posPBF with AG heterozygotes of rs731236, but it did not reach the level of statistical significance, OR = 0.07, *p* = 0.068. For participants with a negPBF with the CC of rs7975232 or AA of rs731236, the risk of bone fractures was lower and, in the case of the AG of rs731236, it was higher; however, that effect was only close to the level of statistical significance.

The bone fracture risk was significantly higher for the group of patients with the CC homozygotes of rs1544410: OR = 30.00, *p* < 0.05, but that applied only to the patients with posPBF. Regarding the patients with negPBF, the opposite was true, i.e., the bone fracture risk was significantly lower in the group of patients with the CC of rs1544410: OR = 0.56, *p* < 0.05. The bone fracture risk was significantly lower in the group of patients with the CT rs1544410, OR = 0.05, *p* < 0.05, but only in those with posPBF. For patients with negPBF, the relationship between CT rs1544410 and bone fracture prevalence rates in the patients’ medical history was not statistically significant: OR = 1.56, *p* > 0.05.

The interactions involving the AC rs7975232 heterozygote polymorphism and other clinical factors were also subjected to the simple effects analyses. For the continuous variables, their body weight and BMI median split data were used to maximize the statistical power for simple effect detection. The median body weight and BMI values were equal to 75.0 kg and 30.86, respectively.

**Figure 6:**
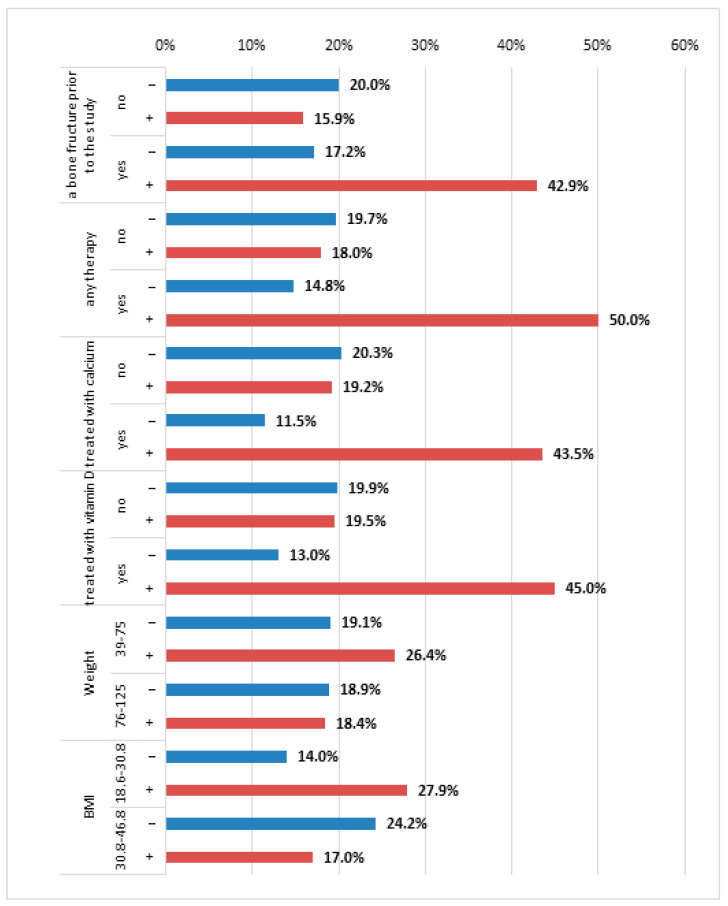
The distribution of bone fractures in the last 10 years depending on the presence of AC heterozygotes of rs7975232. Note: the figure depicts in what percentages of participants with AC heterozygotes of rs7975232 there was a bone fracture in patients’ medical history depending on other factors like BMI, weight, vitamin D intake, etc. Legend: “+” AC heterozygotes of rs7975232present; “−“ AC heterozygotes of rs7975232 absent.

The bone fracture risk was significantly higher in the group of patients with AC rs7975232 heterozygotes, OR = 3.61, *p* < 0.01, but only in those with bone fracture(s) prior to the study (see Figure 6).

Regarding patients without bone fracture incidents before the study, the relationship between AC rs7975232 heterozygotes and bone fracture prevalence rates in the patients’ medical history records was not statistically significant: OR = *0*.40, *p* > 0.05. The bone fracture risk was also significantly higher in the group of patients with AC rs7975232 heterozygotes, OR = 5.75, *p* < 0.05, if they were also simultaneously treated with any therapy. Regarding the patients not being treated with any therapy, the relationship between the AC genotype of rs7975232 and bone fracture prevalence rates in the patients’ medical history records was not statistically significant, OR = 0.70, *p* > 0.05. The same pattern was observed depending on whether the patients were treated with calcium or vitamin D. The bone fracture risk was significantly higher in the group of patients with the AC genotype of rs7975232, OR = 5.90, *p* < 0.05, if they were also treated with calcium. The bone fracture risk was significantly higher in the group of patients with AC rs7975232 heterozygotes, OR = 5.46, *p* < 0.05, if they also received vitamin D.

The bone fracture risk was significantly higher in the group with AC rs7975232 heterozygotes, OR = 2.38, *p* < 0.05, if their BMI values were within the range of 18.6–30.8, i.e., below the median value. For the patients with BMI values above the median value, the correlation was not statistically significant.

## 4. Discussion

The null hypothesis that the population is at the HWE was not rejected. This applies to all three polymorphisms examined.

There were no direct correlations between fracture risk and VDR polymorphisms. However, the study revealed that fracture risk is connected with the genetic pattern and its expression is dependent on some clinical factors. Its protective or harmful role may be connected with paternal fracture history, prior fracture(s), BMI value, any osteoporotic treatment or calcium/vit. D supplementation.

The study, as mentioned before, was carried out as a follow-up part of a large epidemiological research project on postmenopausal women. The main objective of the study was to answer the question of whether the selected VDR gene polymorphism or a set of them could increase the 10-year fracture risk in the postmenopausal women examined. There are well-known fracture risk calculators, such as FRAX [24], Garvan [25] or POL-RISK [26] calculators. Both FRAX and Garvan can be used without the DXA being performed. The fracture risk is based on clinical factors. All of those risk factors were analyzed in our study as well. There were no statistically significant differences detected in the 10-year fracture risk from the aspect of polymorphism variants. That observation may suggest that the genotyping of the selected polymorphisms may be insufficient to identify groups with a higher fracture risk. Therefore, the VDR polymorphisms were analyzed in combination with clinical factors.

Wu et al. [27] undertook to assess bone fracture risk in postmenopausal women both by the FRAX calculator without DXA and on the basis of genetic risk factors. That included 14 fracture-associated SNPs, discovered in a large genome-wide meta-analysis [28]. The authors of the study showed the 10-year fracture risk, assessed by the FRAX calculator, to have been overestimated, compared to genetic profiling. Their work was an important signal, showing that not only clinical factors, but also genetic profiling should be taken into account in bone fracture risk assessments. Nevertheless, the SNPs analyzed in the cited study did not include the SNPs assessed in our study, thus excluding any possibility of direct comparisons [27,29].

The rs731236 (TaqI) polymorphism, similarly as in our study, was analyzed in a study by Senosi [30]. The focus group included patients suffering from rheumatoid arthritis. The authors did not find any correlation between the VDR polymorphisms and rheumatoid arthritis, and then concluded that the polymorphisms did not affect bone fracture risk (gene polymorphisms do not change the risk of bone fractures, which could be the case if they influenced RA activity and thus the risk of developing osteoporosis).

The observations from the study by Mondockova et al., conducted on 356 postmenopausal Caucasian women, seem to be contrary to our results. The authors found, in a retrospective analysis, that the ApaI, TaqI and BsmI genotypes increased the risk of spinal, radial or total fractures [31]. The fracture incidence analysis was based on X-rays which helped avoid clinically silent fractures. Nevertheless, other research projects assume that there is no connection between VDR polymorphisms and the fracture incidence [16,32].

When the study group was divided into two categories (with a co-occurring CC of rs7975232, AA of rs731236 and CC of rs1544410 vs. AC of rs7975232, AG of rs731236 and CT of rs1544410), significant changes were found in the aspect of 10-year fracture records, depending on the bone fracture rates in the medical history of the patients’ fathers. The configuration of CC rs7975232, AA rs731236 and CC rs1544410 homozygotes seemed to increase the fracture risk in women whose fathers suffered from a bone fracture. On the other hand, participants with the CT of rs1544410 with posPBF were at a lower fracture risk. No similar connection was identified between the fracture risk and maternal fracture history. The influence of parental fracture history on fracture risk is a known and well-confirmed factor [33,34]. It proves the influence of genetic factors on the risk of fractures. A new observation in our study was that the association of genetic factors with the current bone fracture risk occurred only in patients with posPBF [35]. There are few works dedicated to this issue. In The Rancho Bernardo Study, conducted on a group of 1477 older volunteers (877 of them were women), the authors assessed the impact of parental and sibling history on the bone status of the study participants. In that group of women, a significant relationship was found between the paternal history of fractures and lumbar spine BMD, but no such relationship was found for maternal fractures [36]. That was probably the first study to examine the effects of maternal and paternal fracture history in separate approaches. The authors suggested that their observations could indicate not only a hormonal, but also a genetic background for the development of osteoporosis. Their conclusions, combined with the results of our analyses, seem to confirm the observation.

In another study, Yang et al. [37] used population-based administrative healthcare data for the province of Manitoba, Canada, and identified 255,512 offsprings with a linkage to at least one parent. Interestingly enough, that project included both hip and other major osteoporotic fractures (MOFs). The authors of the study showed a significantly higher risk of fractures in the offspring of parents with a positive history of fractures. That observation does not fully correspond to our results but brings some further implications: the risk of fractures in the offspring increased with the number of parents (1 or 2) with MOF, the number of these fractures and when both a hip fracture and another MOF occurred in the parents. This phenomenon can be referred to as the ‘dose effect’. In our study, the parental history of any fractures was analyzed (hip, spine, femur, arm, forearm, lower leg, rib, foot) also from the aspect of the number of fractures, but no similar observation was derived.

Our study revealed that the risk of a bone fracture is significantly higher in the group of heterozygotes of AC rs7975232 if they were treated with calcium and/or vitamin D. It may be assumed that patients already diagnosed with osteoporosis/osteopenia at the beginning of the follow up started or continued supplementation with vit. D and/or calcium preparations, and that the higher fracture risk was due to the initially poor bone parameters. Nevertheless, that group of patients differed significantly from those without supplementation. This can be explained either by resistance to therapy or disorders in vitamin D metabolism. One of the causes could be the presence of a heterozygous allele AC genotype for the rs7975232 polymorphism in the VDR gene, which may have affected the poorer absorption of vitamin D and thus contributed to the resistance to it.

Moreover, the 10-year bone fracture risk was significantly higher, again, in the group of heterozygotes of AC rs7975232, if their BMI values were within the categories of normal weight or overweight, but not obesity. Osteoporosis and obesity are nowadays widely studied from the aspect of a common genetic pathway. There were many candidate genes examined [38,39,40]. In a study by Chang et al. [41] on a group of 168 women aged 24–75 years, the relationship between VDR polymorphisms (BsmI, TaqI and ApaI) was assessed, among others, from the aspect of impaired calcium absorption in the intestines in relation to body weight changes. It was shown that during energy restriction, genetic differences (in the case of this study, in terms of BsmI) may have exacerbated the expected decrease in calcium absorption. No such relationships were observed for ApaI. The authors of the study explained the relationship between reduced calcium absorption and body weight reduction by an increase in cortisol levels, a decrease in estradiol levels and a decrease in the supply of fat in the diet.

To the best of our knowledge, our study on the 10-year fracture risk from the aspect of genotype profiling was the first one to raise the issue. Other advantages of the study included the large study group and the long observation period. All the participants were at a stage after menopause, which excluded any possibility of a hormonal impact on bone metabolism; on the other hand, the focus group was the most exposed to fractures. The database included a large amount of information about both modifiable and non-modifiable fracture risk factors. It was also one of the research projects analyzing the risk of bone fractures in terms of fracture history in parents, separately for the mother and father.

A main limitation in the study was the inability to verify the number of fractures in the available medical records or imaging studies; the fracture information was based on declarative reports, with an obvious risk to disregard clinically silent fractures in the analysis. Another limitation influencing the final number of the study participants was technical, resulting from contact loss in the case of some participants. Taking into account these study limitations, it should still be emphasized that the number of fractures during the 10-year observation was sufficient to form final conclusions. It may be assumed that these relationships between the selected VDR polymorphisms and bone fracture rates would be even stronger after asymptomatic fracture evaluation.

## 5. Conclusions

The polymorphisms of rs1544410, rs7975232 and rs731236 are connected with a 10-year fracture risk in postmenopausal women, but their influence does not seem to be direct. It should be considered together with other co-existing clinical factors, especially paternal fracture history, prior fracture, BMI value, any osteoporotic treatment or calcium/vit. D supplementation. Further research in this area of genetic profiling may, in the future, make it possible to identify genetic markers which could, for example, precisely identify therapy responders.

## Figures and Tables

**Table 1:** Descriptive characteristics of the study group (*n* = 358).

Parameter	Mean	Min.	Max.	SD
Age [years]	65.21	55	84	6.91
BMI [kg/m^2^]	31.23	18.61	46.8	5.52
Deliveries	2.46	0	12	1.39
Pregnancies	2.7	0	14	1.55
Medical characteristics of the study group				
Variable	*n*	%		
Rheumatoid arthritis	18	5.0		
Diabetes type 1	13	3.6		
Diabetes type 2	51	14.2		
Treated with steroids	15	4.2		
Treated with any antiosteoporotic therapy	51	14.2		
Treated with alendronate	19	5.3		
Treated with calcitonin	4	1.1		
Calcium treatment/supplementation	49	13.7		
Vitamin D treatment/supplementation	43	12.0		
Positive history of cigarette smoking	36	10		
Chronic alcohol use	3	0.8		
Family history of fracture				
Parental history of bone fracture	100	27.9		
Maternal history of bone fracture	84	23.5		
Paternal history of bone fracture	22	6.1		

Legend: *n*—number of participants; %—sample percentage; treated with steroids—oral glucocorticoids for more than 3 months at a dose of prednisolone of 5 mg daily or more (or equivalent doses of other glucocorticoids); chronic alcohol use—3 or more units of alcohol per day.

**Table 2:** Genotype and allele frequencies of the analyzed VDR gene polymorphisms.

SNP	Genotype	*n* (%)	Allele	*n* (%)	HWE *p*-Value
rs7975232	AA	92 (25.7%)	A	358 (50.0%)	0.597
	AC	174 (48.6%)	C	358 (50.0%)	
	CC	92 (25.7%)			
	AA + AC	266 (74.3%)			
	AC + CC	266 (74.3%)			
rs731236	AA	152 (42.5%)	A	457 (63.8%)	0.159
	AG	153 (42.7%)	G	259 (36.2%)	
	GG	53 (14.8%)			
	AA + AG	305 (85.2%)			
	AG + GG	206 (57.5%)			
rs1544410	CC	144 (40.2%)	C	449 (62.7%)	0.467
	CT	161 (45.0%)	T	267 (37.3%)	
	TT	53 (14.8%)			
	CC + CT	305 (85.2%)			
	CT + TT	214 (59.8%)			

**Table 3:** Relationships between polymorphism variants, clinical characteristics and bone fractures.

		Group 1		rs7975232				rs731236				rs1544410			
Medical Characteristic	Effect	vs. Group 2		CC		AC		AA		AG		CC		CT	
		*OR*	*p*	*OR*	*p*	*OR*	*p*	*OR*	*p*	*OR*	*p*	*OR*	*p*	*OR*	*p*
A bone fracture	main	1.36	0.266	0.68	0.232	1.32	0.298	0.77	0.340	1.33	0.275	0.74	0.266	1.29	0.331
prior to the study	interaction	0.43	0.138	1.03	0.962	**4.78**	**0.005**	2.97	0.055	0.61	0.370	2.34	0.138	1.17	0.772
Treated with	main	1.47	0.165	0.67	0.204	1.24	0.411	0.71	0.212	1.38	0.223	0.68	0.165	1.33	0.281
any therapy	interaction	1.26	0.738	0.60	0.529	**6.44**	**0.012**	1.20	0.790	1.50	0.553	0.79	0.738	2.50	0.183
Treated with	main	1.45	0.178	0.68	0.221	1.24	0.419	0.73	0.235	1.37	0.234	0.69	0.178	1.33	0.284
calcium	interaction	1.29	0.725	0.48	0.418	**6.30**	**0.021**	1.60	0.511	1.13	0.863	0.77	0.725	2.67	0.178
Treated with	main	1.45	0.177	0.68	0.223	1.24	0.416	0.73	0.237	1.36	0.245	0.69	0.177	1.32	0.293
vitamin D	interaction	1.04	0.963	0.57	0.546	**5.59**	**0.035**	2.15	0.304	0.69	0.619	0.97	0.963	1.72	0.468
Weight	main	1.42	0.198	0.67	0.209	1.25	0.397	0.72	0.227	1.37	0.229	0.70	0.198	1.32	0.295
	interaction	1.02	0.265	1.01	0.588	**0.96**	**0.024**	1.00	0.982	0.98	0.388	0.98	0.265	1.01	0.708
BMI	main	1.41	0.205	0.68	0.227	1.23	0.424	0.73	0.242	1.36	0.246	0.71	0.205	1.29	0.324
	interaction	1.04	0.431	1.03	0.623	**0.90**	**0.026**	1.01	0.848	0.96	0.347	0.96	0.431	1.01	0.898
Bone fractures	main	1.41	0.211	0.69	0.236	1.23	0.438	0.73	0.249	1.35	0.256	0.71	0.211	1.29	0.341
in father’s medical history	interaction	**0.02**	**0.004**	**45.36**	**0.007**	0.21	0.147	**31.41**	**0.009**	**0.07**	**0.032**	**53.49**	**0.004**	**0.03**	**0.010**

Legend: *OR*—odds ratio; *p*—*p*-value; BMI—body mass index; treated with any therapy—any osteoporotic therapy. Statistically significant effects are marked with a bold font. Based on the analysis of the logistic regression, no statistically significant main effects of polymorphism variants were detected, which means that bone fractures were not explained by the polymorphisms only. However, there were 12 statistically significant relationship effects. In order to interpret the interactions detected, a simple effects analysis was performed.

## Data Availability

Data are available on special request from the corresponding author.

## Supplementary Materials

The following supporting information can be downloaded at: https://www.mdpi.com/article/10.3390/nu16234146/s1, Table S1. Single nucleotide variations—characteristcs.
