# Supplementary material for: Bone Fracture Incidence in Postmenopausal Women: Results of a 10 Year Follow Up in a RAC-OST-POL Study of rs1544410, rs7975232 and rs731236 Polymorphisms
Source: Nutrients. 2024 Nov 29;16(23):4146. doi: 10.3390/nu16234146 (PMC11643942; doi:10.3390/nu16234146)
Supplement: Supplementary file 1 [file nutrients-16-04146-s001.zip › nutrients-3309063-supplementary.pdf]

Supplementary Table S1: Single nucleotide variations—characteristics.

Single nucleotide variations:	Ref	Alt	Position	GMAF
rs1544410 (BsmI)	C	T	chr12:4784605	0.2649
rs7975232 (ApaI)	C	A	chr12:47845054	0.4959
rs731236 (TaqI)	A	G	chr12:47844974	0.264

Ref—reference allele, Alt—alternate allele, Position—position in the genome according to Homo sapiens (human) genome assembly GRCh38 (hg38_HC) GRCh38.p14 chr 12, GMAF—global minor allele frequency.
